# Antitumor activity of recombinant oncolytic vaccinia virus with human IL2

**DOI:** 10.1515/med-2022-0496

**Published:** 2022-06-17

**Authors:** Liqiong Liu, Huiqun Li, Qinggang Xu, Yan Wu, Dongfeng Chen, Feng Yu

**Affiliations:** Department of Hematology, Huazhong University of Science and Technology Union Shenzhen Hospital, Shenzhen, Guangdong Province, 518052, P. R. China; School of Life Sciences, Jiangsu University, Zhenjiang, 212013, P. R. China; School of Life Sciences, Jiangsu University, No. 301 Xuefu Road, Zhenjiang, 212013, P. R. China

**Keywords:** thymidine kinase, viral growth factor, human interleukin-2, oncolytic vaccinia, tumor

## Abstract

The tumor microenvironment is highly immunosuppressive. The genetically modified oncolytic vaccinia virus (OVV) is a promising vector for cancer immunotherapy. The aim of the present study was to assess the antitumor effects of human interleukin-2 (hIL2)-armed OVV *in vitro*. The hIL2 gene was inserted into a thymidine kinase and the viral growth factor double deleted oncolytic VV (VVDD) to generate recombinant hIL2-armed OVV (rVVDD-hIL2). Viral replication capacity in A549 cells was quantified by plaque titration on CV-1 cells. Production of hIL2 in cancer cells infected by rVVDD-hIL2 was measured by enzyme-linked immunosorbent assay. Finally, 3-(4,5-dimethylthiazol-2-yl)-5-(3-arboxymethoxyphenyl)-2-(4-sulfophenyl)-2H-tetrazolium, inner salt (MTS) assay was performed to assess the antitumor effects of rVVDD-hIL2. The results showed that rVVDD-hIL2 viral particles expressed increasing levels of hIL2 in human and murine cancer cell lines with growing multiplicities of infection (MOIs). The insertion of the hIL2 gene did not impair the replication capacity of VV, and the rVVDD-hIL2 virus killed cancer cells efficaciously. The lytic effects of the recombinant oncolytic virus on tumor cells increased with the growing MOIs. In conclusion, these findings suggest that hIL2-armed VVDD effectively infects and lyses tumor cells, with high expression of hIL2.

## 1 Introduction

Cancer is one of the leading causes of rising global mortality [1]. The immune system greatly impacts the inhibition of proliferation and metastasis of cancer cells [2]. Indeed, the immune score based on the number of tumor-infiltrating CD3^+^ CD8^+^ T cells can even predict patient prognosis [3]. However, traditional treatments, including surgery, chemotherapy, and radiotherapy, inhibit cancer cells without sparing normal cells in patients, thereby further hampering the patients’ immunity. Although immunotherapy and targeted therapy have been proposed as promising treatment options for cancer to reverse the abovementioned defects, limited success in antitumor therapy has been achieved by available immunotherapies, including adoptive immune cell therapy, cancer vaccines, and check point blockade [4]. Tumors also affect the immune system by attenuating the effects of both cellular and humoral immunities [5]. Taken together, the tumor microenvironment (TME) in the setting of advanced-stage cancer is highly immunosuppressive, which impairs the immune response to cancer [6].

Recent studies have suggested that oncolytic viruses (OVs) can selectively infect and kill cancer cells while favorably modulating the TME [7]. The antitumor (and antiviral) immunity is elicited by OVs as a consequence of improved antigen cross-priming and recruitment of immune cells into the TME [7,8]. The cytokine IL2, acting as a very well-known activation factor for the proliferation and differentiation of T and NK cells, enhances antibody-dependent cell-mediated cytotoxicity [9,10]. IL2 represents the first effective immunotherapeutic agent, benefiting patients with metastatic melanoma and advanced non-Hodgkin’s lymphomas [11–14]. However, whether IL2-armed oncolytic vaccinia virus (OVV) could exert antitumor effects remains largely unknown. Therefore, this study aimed to generate a double deleted recombinant OVV armed with the human IL2 gene and to determine its expression levels of human interleukin-2 (hIL2) as well as oncolytic effects in both human and murine cancer cell lines. We found that hIL2-armed double deleted vaccinia virus (VV) could effectively infect and lyse cancer cells, with high expression of hIL2.

## 2 Materials and methods

### 2.1 Cell lines and virus

Human osteosarcoma HUTK-143B, human cervical cancer HelaS3, African green monkey kidney CV-1, human breast cancer MCF-7, human lung adenocarcinoma A549, and mouse colon adenocarcinoma MC38 and CT26 cells were obtained from the American Type Culture Collection (ATCC, Rockville, MD, USA). All cell lines were cultured in recommended media supplemented with 10% fetal bovine serum. vSC20 virus, rVVDD-GFP, and the recombinant plasmids pUC57-hIL2 and pSEL2N1 are kept in our laboratory.

### 2.2 Generation of the recombinant plasmid pSEL2N1-hIL2

The sequence of the hIL2 gene was amplified from the pUC57-hIL2 plasmid with the primers PF17R-hIL2-F (GGTATCTTGCGGGATATCAAAAATTGAAATTTTATTTTTTTTTTTTGGAATATAAATAAATGTACAGGATGCAACTCCTGTC) and PF17R-hIL2-R (GTCAAAGCATCATCTCAACACTGACTTGAGCGGCCGCGACTCTAGATCATAA), synthesized by Sangon (Shanghai, China). Restriction sites for *Eco*R V and *Not* I (underlined) were introduced in the hIL2 gene flank using the above primer pair. The polymerase chain reaction (PCR) program for hIL2 amplification was: 98°C for 20 s; 30 cycles of 98°C for 15 s, 60°C for 15 s, and 72°C for 15 s; 72°C for 5 min. The obtained hIL2 DNA was digested with *Eco*R V and *Not* I (NEB, USA) and introduced into the linear plasmid DNA pSEL2N1 cut with the same enzymes to yield the new plasmid pSEL2N1-hIL2.

#### 2.2.1 Generation of a thymidine kinase gene and vaccinia growth factor gene double deleted virus

vSC20, a VV with a depleted viral growth factor gene, was used as the parental virus. Homologous recombination was carried out by transfection of the recombinant plasmid pSEL2N1-hIL2 into CV-1 cells, which were infected with the parental vSC20 VV. Screening and purification of recombinant oncolytic vaccinia were carried out by virus plaque formation on soft agar using HUTK-143B cells. Single plaques were picked and amplified in CV-1 cells. The levels of hIL2 were determined by enzyme-linked immunosorbent assay (ELISA) in supernatants collected from CV-1 cells infected with a single plaque. hIL2-positive plaques were further purified by the plaque assay. After five rounds of screening and purification, a purified recombinant virus termed rVVDD-hIL2 was obtained. The purified recombinant VV rVVDD-hIL2 was amplified in HeLaS3 cells and titrated in CV-1 cells.

### 2.3 Replication capacity assay

A549 cells were plated at 1.5 × 10^5^ per well in 12-well plates. rVVDD-hIL2, rVVDD-GFP, and vSC20 were added to each well at an MOI of 0.01 and incubated at room temperature. Thirty minutes later, the infection medium was changed to a fresh medium. Supernatants and cells were collected at the indicated times. After a single freeze–thaw cycle, the virus was quantified by plaque titration on CV-1 cells as described previously [8], in triplicate.

### 2.4 hIL2 expression of recombinant rVVDD-hIL2

Human cancer MCF-7 and A549 and murine cancer MC38 and CT26 cells were seeded into 96-well plates at a density of 1 × 10^4^ cells per well. At 90% confluence, the cells were infected at different MOIs of 0, 0.01, 0.1, 1, and 10, respectively. After culture in an incubator at 37°C with 5% CO_2_ for 48 h, the supernatants were collected for ELISA with the IL2 ELISA kit (BD, USA) as directed by the manufacturer.

### 2.5 MTS assay

The MTS assay was performed to assess the antitumor effect of hIL2-armed recombinant oncolytic vaccinia. After collecting the supernatants, MCF-7, A549, MC38, and CT26 cells remaining in 96-well plates were subjected to the MTS assay, with the MTS Kit (Promega, USA) according to the manufacturer’s instructions.

### 2.6 Statistical analysis

Statistical analysis was performed with SPSS version 19.0 (SPSS Inc., Chicago, IL, USA). Data are mean ± standard deviation (SD). Student’s *t*-test was used to analyze the difference between the two groups. One-Way ANOVA analysis of variance was applied to compare multiple groups. Statistical significance was set at *P* < 0.05.

## 3 Results

### 3.1 Generation of rVVDD-hIL2

**Figure 1:**
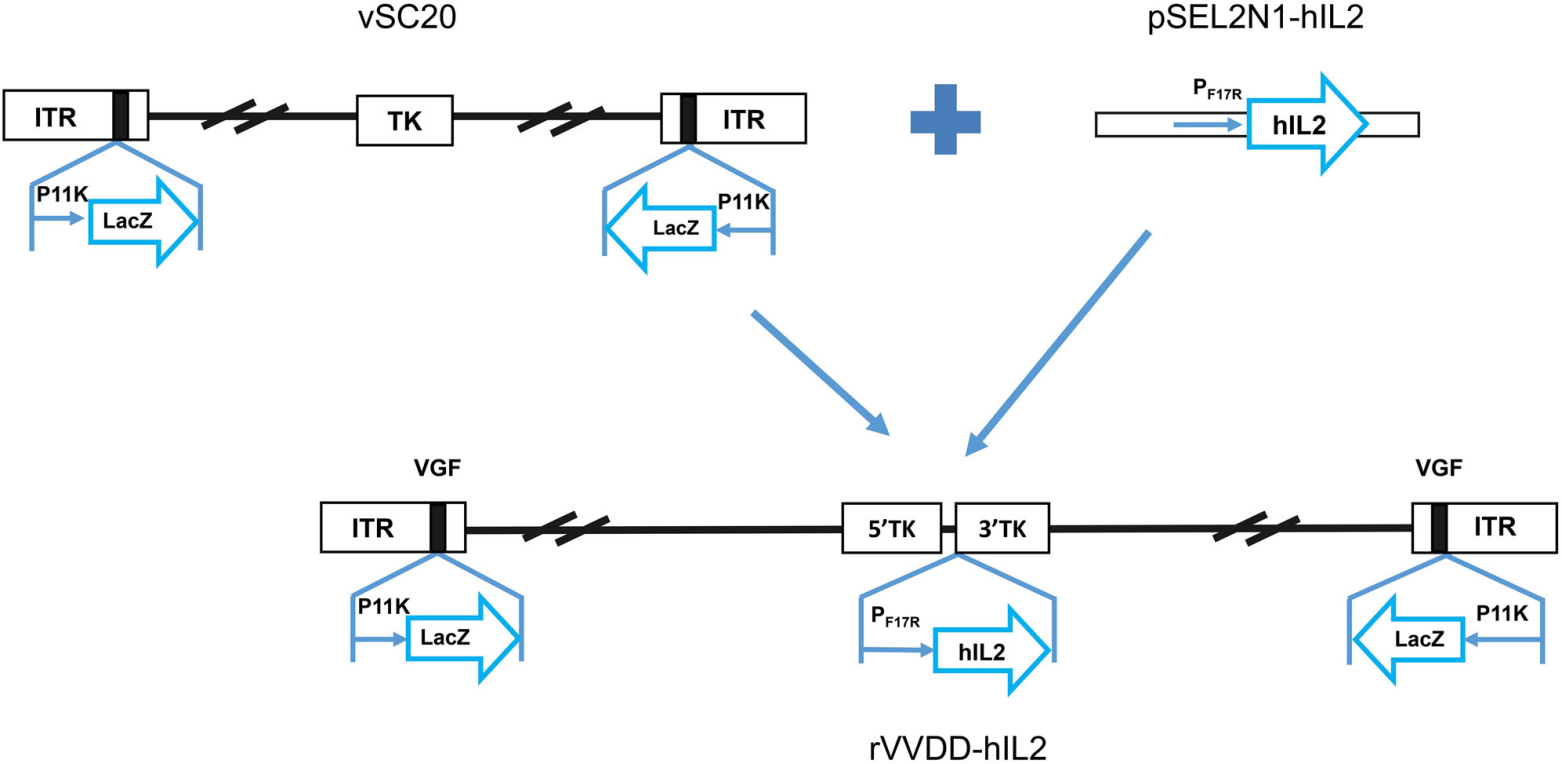
Schematic structure of rVVDD-hIL2 encoding a human IL2 expression cassette.

**Figure 2:**
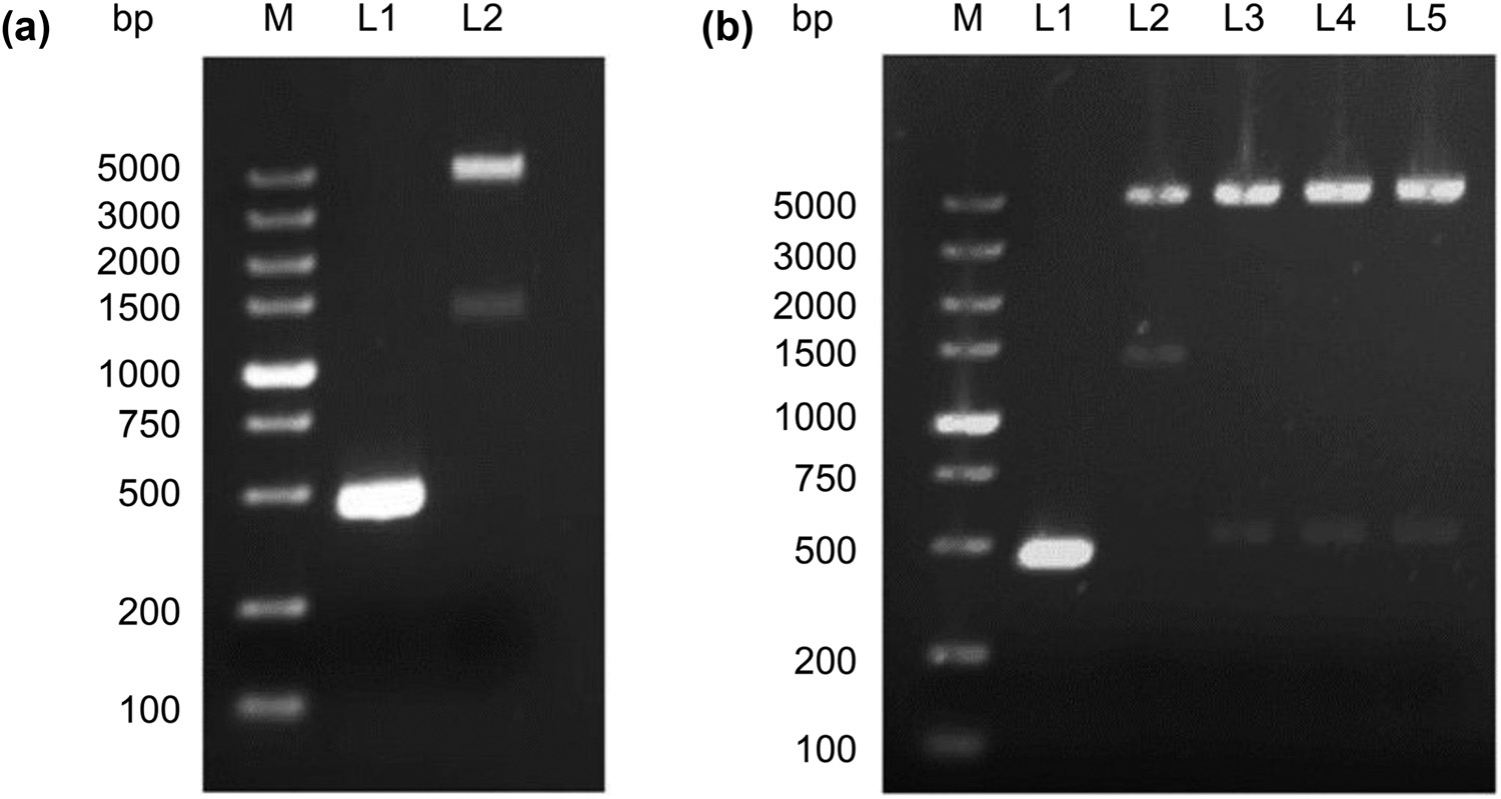
Construction of the pSEL2N1-hIL2 recombinant plasmid. (a) Preparation of the backbone DNA and inserted PCR fragments. M, DL5000 DNA ladder; L1, PCR product of hIL2; L2, pSEL2N1 vector digested with *Eco*R V and *Not* I. (b) Screening of positive clones by restriction endonuclease digestion. M, DL5000 ladder; L1, PCR product of IL2; L2∼5, recombinant plasmids identified by *Eco*R V and *Not* I digestion.

rVVDD-hIL2 was constructed by insertion of the hIL2 gene into the vSC20 virus using homologous recombination as illustrated in Figure 1. The hIL2 gene was amplified from the pUC57-hIL2 plasmid by PCR, and the target gene fragment was obtained by gel recovery (Figure 2b). After double enzyme digestion, hIL2 was inserted into pSEL2N1 (Figure 1). The positive recombinant plasmid pSEL2N1-hIL2 was identified by double enzyme digestion (Figure 2b) and further confirmed by sequencing.

### 3.2 Expression of hIL2 in cancer cells

**Figure 3:**
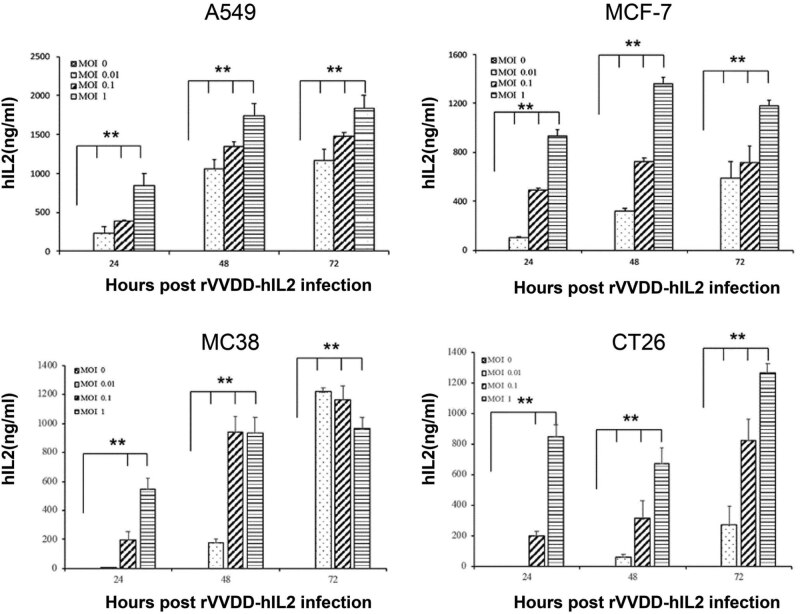
IL2 secretion levels in different cancer cell lines infected with rVVDD-hIL2. Cancer cells were infected with rVVDD-hIL2 at increasing MOIs (0, 0.01, 0.1, and 1), and cell culture supernatants were collected at 24, 48, and 72 h and subjected to ELISA. Human cancer MCF-7 and A549 and murine tumor MC38 and CT26 cells were analyzed. **P < 0.01 compared to the MOI of 0. Data were derived from two independent experiments. All comparisons were analyzed by the student’s *t*-test.

The expression levels of hIL2 in cancer MCF-7, A549, MC38, and CT26 cells infected with rVVDD-hIL2 at increasing MOIs (0, 0.01, 0.1, and 1) were determined. Cell supernatants were collected at different time points (24, 48, and 72 h) and subjected to ELISA. The results showed that hIL2 amounts increased in these cells with the MOI of rVVDD-hIL2, in a time-dependent manner (Figure 3). MC38 cells constitute a good mouse cell model for OVV due to their fast growth capacity and sensitivity to VV oncolysis. In this study, hIL2 expression in MC38 cells was inversely correlated with MOI at 72 h post-infection, mainly because most cells were lysed at MOIs of 0.1 and 1.

#### 3.2.1 hIL2 insertion does not impair the replication capacity of VVs

**Figure 4:**
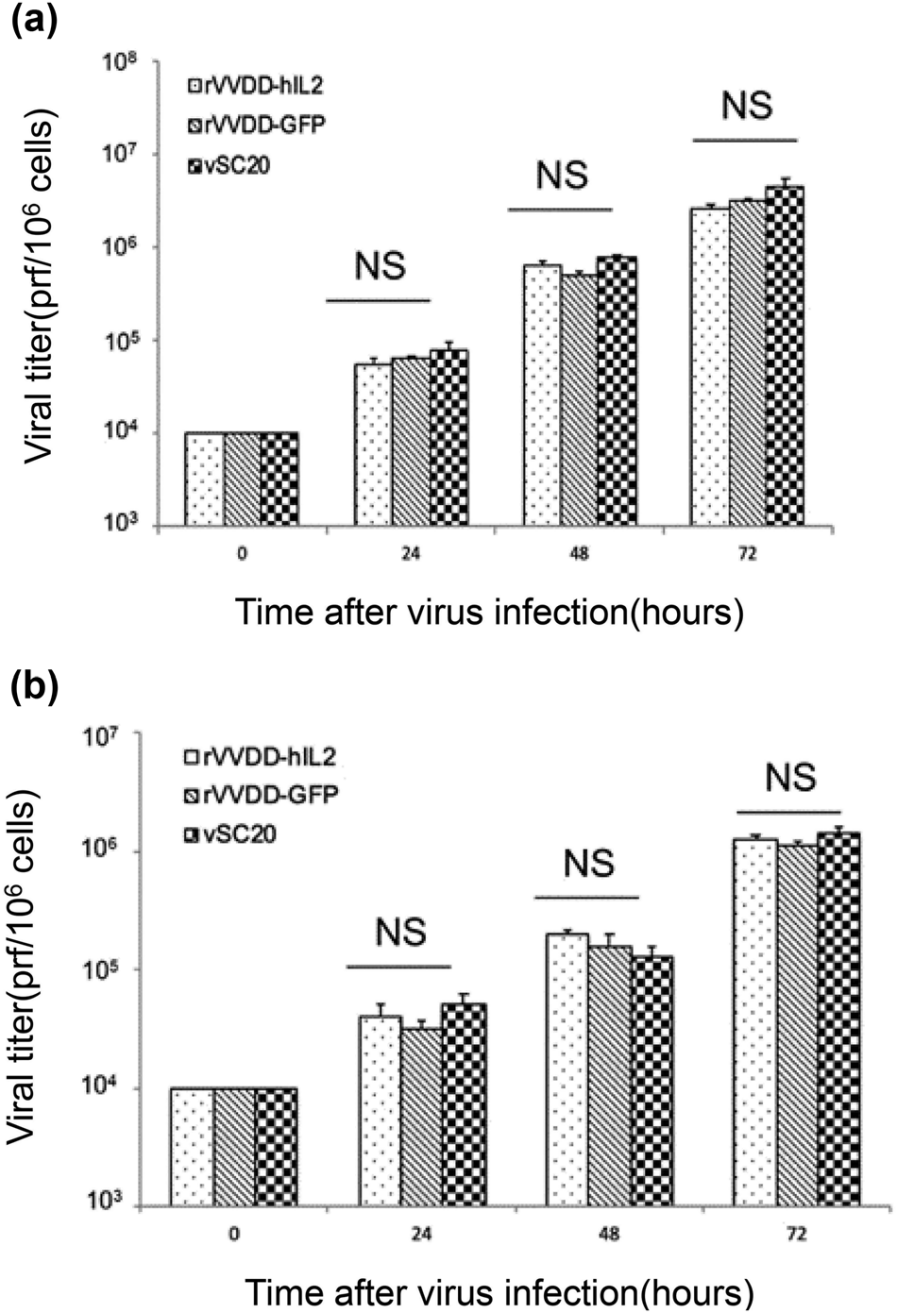
Replication efficiency of rVVDD-hIL2 in tumor cell lines. (a) Viral titers in a human tumor cell line. A549 ells were infected with vSC20 or its derivatives rVVDD-hIL2 and rVVDD-GFP, at an MOI of 0.01 and harvested at the indicated times post-infection. Viral titers were determined in CV-1 cells. (b) Viral titers in a mouse tumor cell line. MC38 cells were infected with the same viruses as A549 cells, and viral titers were also tested in CV-1 cells. NS, non-significant (*P* > 0.05). All comparisons were analyzed by one-way analysis of variance.

To determine whether hIL2 insertion affects the replication ability of rVVDD-hIL2, human A549 and murine MC38 cells were infected with rVVDD-hIL2, rVVDD-GFP, and the parental vSC20 virus at an MOI of 0.01. Infection of A549 cells *in vitro* produced comparable amounts of virus at three different time points (Figure 4a). Consistently, MC38 cells yielded similar amounts of virus particles at each time point (Figure 4b). Therefore, human IL2 insertion did not interfere with VV replication.

### 3.3 rVVDD-hIL2 efficiently suppresses cancer cell growth *in vitro*

**Figure 5:**
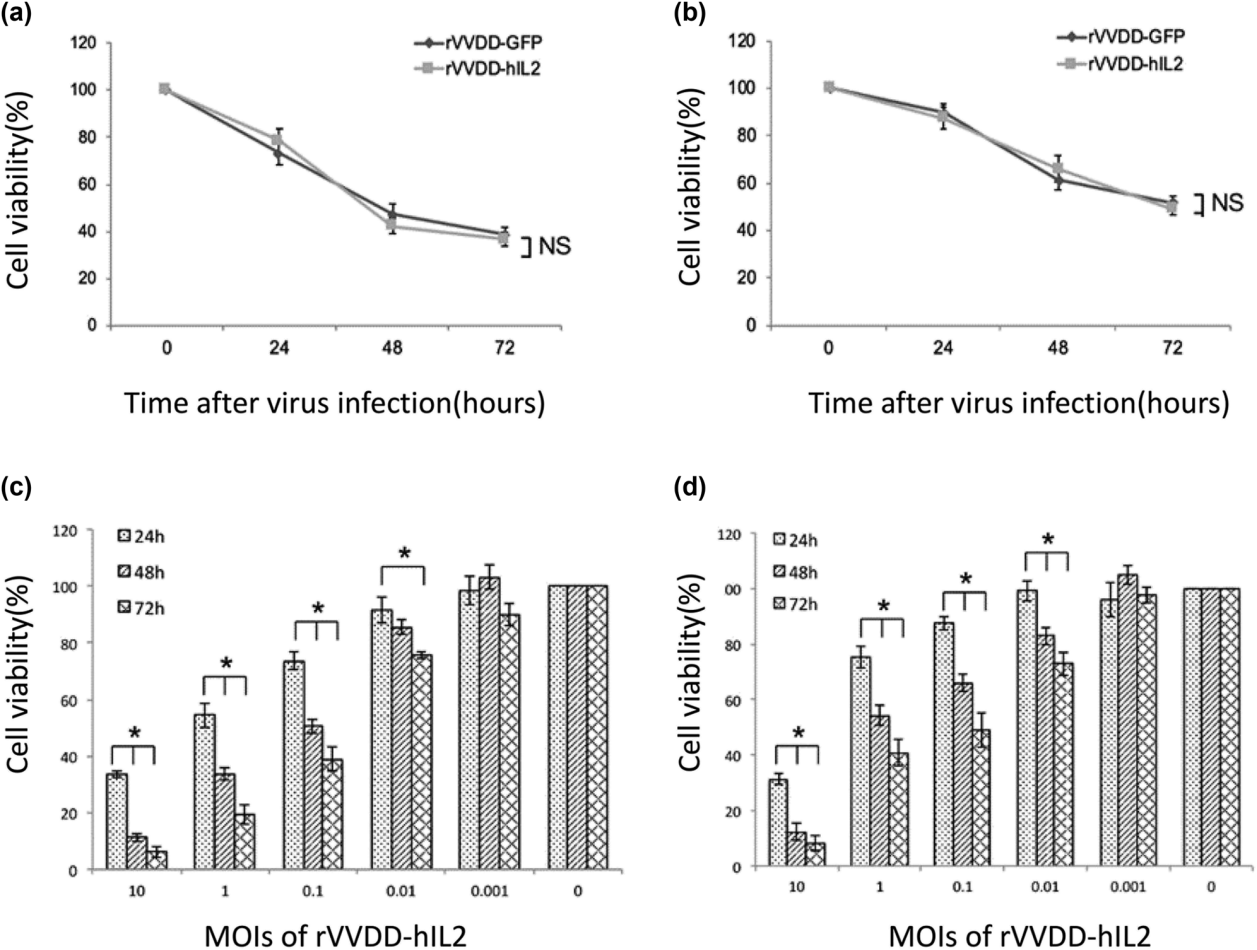
Oncolytic activities of rVVDD-hIL2 an rVVDD-GFP in tumor cell lines. Human lung carcinoma A549 (a) and murine colon tumor MC38 (b) cells were infected with rVVDD-hIL2 and rVVDD-GFP at an MOI of 0.1, respectively. Cell viability was determined by the MTS assay at the indicated time points (rVVDD-hIL2 vs. rVVDD-GFP, *P* > 0.05, NS). (c and d) A549 and MC38 cells were infected with rVVDD-hIL2 at increasing doses (MOIs of 0.001, 0.01, 0.1, 1, and 10), and cell viability was measured by the MTS assay at the indicated times post-infection. *P < 0.05 compared to the cell viability at the time point of 24 h. Data were derived from three independent experiments. All comparisons were analyzed by the student’s *t*-test.

To further compare rVVDD-hIL2 and rVVDD-GFP for oncolytic ability, A549 and MC38 cells were infected with rVVDD-hIL2 or rVVDD-GFP at an MOI of 0.1. Tumor cell viability was determined by the MTS assay. At all time points, rVVDD-hIL2 and rVVDD-GFP had similar cytolytic effects (Figure 5a and b). Next, we determined the cytotoxicity of rVVDD-hIL2 by infecting A549 and MC38 cells with increasing MOIs. The growth rates of both A549 and MC38 cells were effectively suppressed, in an MOI-dependent fashion (Figure 5c and d). Jointly, these findings indicated that rVVDD-hIL2 had strong antitumor activity.

## 4 Discussion

This study demonstrated that human IL2-armed double deleted VV effectively infects and lyses cancer cells, with the high expression of human IL2. The TME is highly immunosuppressive, impairing systemic antitumor immunity, which leads to unlimited tumor proliferation [15]. Immunotherapy has shown great promise in cancer treatment [16–18], and various strategies have been investigated to promote the antitumor immunity, including enhancing tumor immunogenicity and the infiltration efficiency of tumor-specific T cells [19–22]. OVs have also demonstrated promising results in the treatment of cancer [15,23]. For example, VV has exhibited inspiring results with broad-spectrum infectivity and tumor tropism, as well as no latent risk of carcinogenic potential in the treatment of cancer because its genomic DNA is not integrated into host chromosomes [24]. The VVDD has attracted wide attention for excellent selective tumor-killing performance without affecting surrounding normal cells [23,25]. Therefore, VVDD is considered an ideal therapeutic vector for targeted cancer therapy.

Cytokines are molecular messengers allowing immune cell communication and affecting the proliferation and invasion of cancer cells in the TME [26,27]. IL2, a well-known T-cell growth factor approved for the treatment of metastatic melanoma and renal cell carcinoma, promotes the *in situ* recruitment and activation of cytotoxic immunocytes and switches the immunosuppressive TME toward antitumor immune responses [14,28]. However, high-dose IL2 causes multiple adverse effects, such as vascular leak syndrome, while low dose leads to unwanted preferential expansion of regulatory T cells, which might attenuate antitumor immunity [29,30]. Furthermore, systemic administration of IL2, compared with localized delivery (e.g., aerosolized delivery), has more toxicity in patients [31]. To enhance the antitumor activity of cytotoxic immunocytes and diminish the related toxicity, combination therapies using IL2 and other immunotherapeutics as well as appropriate delivery of IL2 have been recommended [32]. Antibodies fused with IL2 directed against tumor-associated antigens have been tested in preclinical models with promising results [12,33]. Oncolytic viruses armed with cytokines modify the immune microenvironment and alter antitumor effects [15,34]. To maintain the activity of IL2 and abrogate its toxicity, a new form of IL2 immunotherapy through the delivery of a cell membrane-bound mouse IL2 by tumor-targeted oncolytic vaccinia was investigated. The VVDD-IL2-RG construct, with a glycosylphosphatidylinositol anchor and a rigid linker, was sufficient to cure the majority of mice with early-stage peritoneal colon cancer, although more advanced disease recurred [34].

However, human and murine IL2 receptors differentially respond to the human-IL2 component of immunocytokines [35]. The humanized immunocytokine hu14.18-IL2 was designed to selectively target malignancies and concentrate human IL2 at the tumor site by fusing human IL2 to the heavy chains of the intact humanized form of an anti-GD2 monoclonal antibody [35]. The fusion protein hu14.18-IL2 exhibited potent antitumor effects with negligible IL2-related toxicity in mouse models, while dose-limiting effects related to IL2 toxicities, which may be due to different binding of the fusion protein to mouse vs. human IL2 receptors [35–39]. Therefore, whether human IL2-armed OVs exhibit different effects on tumors compared to mouse IL2 OVs remains unclear.

As both cellular and humoral immunities are responsible for effective immunological clearance of replicating VV in humans, recombinant IL2 may not always result in an overall increase in therapeutic efficacy. In some cases, IL2 inhibited the viral replication of IL2-armed OV through intracellular components, without activating the interferon-signaling pathway [40]. In this study, we armed the tumor-selective OVV VVDD with the human IL2 gene. The recombinant virus produced high levels of human IL2 in infected tumor cells and suppressed tumor cell growth efficiently, maintaining its replication *in vitro*. It should be mentioned that MC38 cells at 72 h showed a somewhat different pattern of hIL2 expression compared with the other cell lines, with no clear MOI-dependence observed. Such difference deserves further attention in future studies.

Safety and efficacy are the most essential considerations for the clinical application of immunotherapy. Increased IL2 amounts at local tumor sites favor the antitumor response, though increased circulating IL2 induced by the recombinant virus may be toxic. The more efficient the vector, the more viruses infect tumor cells and the more circulating cytokines might be expressed, which might lead to increased toxicity of circulating IL2. Substantial efforts have been made to improve efficacy and safety in this context. Here, the successful construction of rVVDD-hIL2 provides an experimental basis for further modification of oncolytic vaccinia.

This study had limitations, mainly because of its *in vitro* nature. Although mouse and human cancer cells were investigated, the results may not reflect the *in vivo* situation. In future studies, hIL2 expression levels will be assessed both in the tumor milieu and circulatory system in mouse models. In case of high levels of hIL2 found outside the tumor tissue, weaker promoters will be applied to control hIL2 secretion, or effective elements could be added to anchor hIL2 to the tumor’s local site. Ultimately, clinical trials are required to test the effectiveness of this approach after satisfactory data are generated in animal models.

In conclusion, immunotherapy is promising in cancer treatment. The immunosuppressive TME contributes to unlimited tumor proliferation by inhibiting the antitumor activity of the immunity system. Novel strategies are required to moderate immune reactions in the TME. Cytokines *in situ* delivered by oncolytic viruses might enhance the antitumor activity of cytotoxic immunocytes without related toxicity as shown in this study, which should be further confirmed by subsequent *in vivo* assays.
